# Glycolysis hijacked: a novel pathogenic role of lipoprotein(a) in valve disease

**DOI:** 10.1093/ehjopen/oeaf069

**Published:** 2025-06-06

**Authors:** Luisa Weiss, Elena Aikawa

**Affiliations:** Center for Interdisciplinary Cardiovascular Sciences, Division of Cardiovascular Medicine, Brigham and Women’s Hospital, Harvard Medical School, 3 Blackfan Street, Boston, MA 02115, USA; Center for Interdisciplinary Cardiovascular Sciences, Division of Cardiovascular Medicine, Brigham and Women’s Hospital, Harvard Medical School, 3 Blackfan Street, Boston, MA 02115, USA; Center for Excellence in Vascular Biology, Division of Cardiovascular Medicine, Brigham and Women’s Hospital, Harvard Medical School, 77 Avenue Louis Pasteur, Boston, MA 02115, USA

**This editorial refers to ‘Fuelling stenosis: the integral role of PFKFB3-mediated glycolysis in Lp(a)-induced valve inflammation’, by M. Peletier *et al.*  https://doi.org/10.1093/ehjopen/oeaf068.**

Calcific aortic valve disease (CAVD), the most prevalent valve disease in the developed world, is characterized by progressive fibro-calcific remodelling of the valve cusps. Once symptomatic and left untreated, CAVD confers a dismal prognosis; with a 2-year mortality rate of 50%.^1^ The only available treatment is surgical or transcatheter aortic valve replacement, primarily indicated in late-stage disease, imposing the unmet clinical need to uncover novel therapeutic targets to delay or countermand CAVD progression. The translation of novel targets into clinical trials for CAVD, however, is hampered by the largely unpredictable, lengthy, and pathophysiologically heterogeneous progression from asymptomatic aortic valve sclerosis to symptomatic aortic stenosis (AS).

Familial aggregation significantly increases the risk of CAVD development,^2^ suggesting a genetic component in CAVD development. The pioneering genome-wide association study for AS identified *LPA*, the gene encoding for the protein component of lipoprotein(a) [Lp(a)], as the first and currently only monogenic CAVD risk factor.^3^ Lipoprotein(a) is a major carrier of oxidized phospholipids (Ox-PL)^4^ and epidemiological and clinical studies associate plasma levels > 50 mg/dL (125 nmol/L) with faster disease progression.^5^ Indeed, in patients with comparable baseline AS severity, elevated Lp(a) levels (defined as >35 mg/dL) independently associated with haemodynamic disease progression, valve replacement and death.^6^ Discerning the molecular pathways underlying the pathophysiological contribution of Lp(a) will be imperative for the development of targeted pharmacological intervention.

In this issue of *European Heart Journal Open*, Peletier *et al*.^7^ utilize a series of well-designed *in vivo*, *in vitro*, and *ex vivo* experiments to comprehensively unveil Lp(a)-induced metabolic modulation in valvular interstitial cells (VICs), leading to sustained aortic valve inflammation. Initially triggered through nuclear factor kappa-light-chain-enhancer of activated B cells activation, a metabolic shift towards enhanced glycolysis provokes sustained Lp(a)-induced inflammatory responses. Bulk RNA sequencing identified increased levels of 6-phosphofructo-2-kinase/fructose-2,6-biphosphatase 3 (PFKFB3), a potent allosteric activator of the glycolytic enzyme phosphofructokinase-1, in Lp(a)-treated VICs. Targeted inhibition of PFKFB3 with Kan0438757 not only abolished glycolytic activation but also ameliorated inflammatory responses in VICs. While inhibition of PFKFB3-mediated glycolysis has previously been reported to attenuate inflammation and osteogenic differentiation in VICs^8^ and cardiac fibrosis,^9^ this article not only identifies Lp(a) as a critical mediator of glycolytic modulation in early-stage CAVD, but also provides novel insights into the intricate crosstalk of inflammation and metabolism, opening novel avenues for the development of sought after targeted therapies for CAVD.

**Figure 1:**
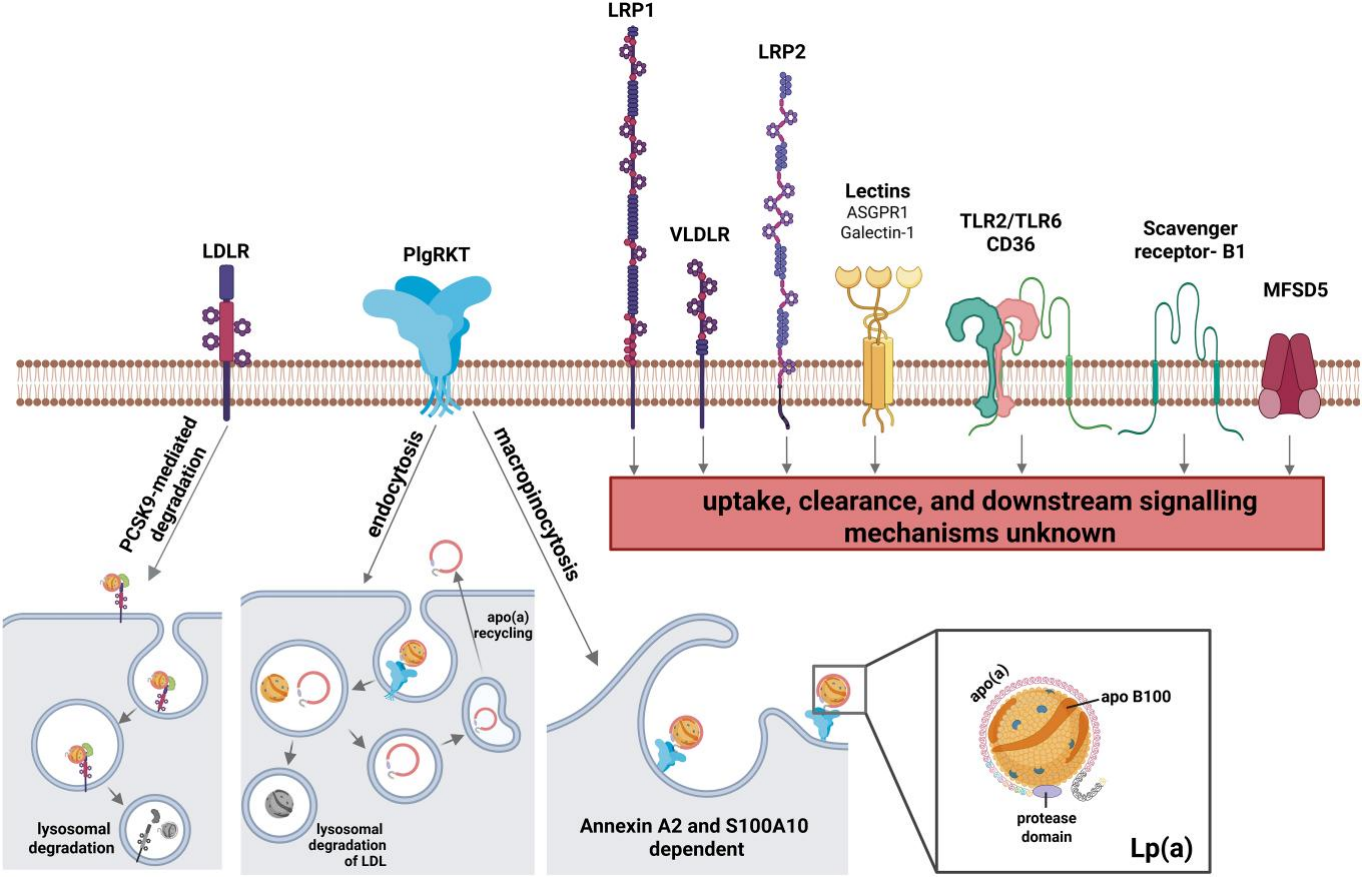
Putative lipoprotein(a) receptors in chronological order of their discovery. Experimental studies suggest direct binding of lipoprotein(a) (-components) to these receptors. However, subsequent uptake, clearance, and/or downstream signalling mechanisms remain largely elusive. ASGPR1, asialoglycoprotein receptor 1; LDLR, LDL receptor; LRP, LDL receptor-related protein; MFSD5, Major Facilitator Superfamily Domain Containing 5; PCSK9, proprotein convertase subtilisin/kexin type 9; PlgRTK, plasminogen receptor with a C-terminal lysine; TLR, toll-like receptor; VLDLR, very LDL receptor.

Of utmost clinical interest is the identification of Lp(a) receptors and uptake mechanisms that can be therapeutically targeted to lower circulating Lp(a) levels. Although primarily indicated for LDL-lowering, proprotein convertase subtilisin/kexin type 9 (PCSK9) inhibition conferred additional benefits by simultaneously lowering Lp(a) levels up to 30% in patients with atherosclerotic cardiovascular disease.^10^ While several receptors have been proposed to bind Lp(a), mainly through its apoB-100 or Ox-PL moieties (*Figure 1*), a specific Lp(a) receptor has not been discovered.^11^ A number of cell type specific downstream signalling mechanisms in response to Lp(a) binding have been suggested,^11^ yet validated uptake mechanisms remain scarce. In this regard, the recent discovery of two independent plasminogen receptor with a C-terminal lysine-mediated Lp(a) internalization and clearance pathways^12,13^ has the potential to extend the repertoire of Lp(a)-lowering therapies in the future. Our group recently discovered Major Facilitator Superfamily Domain Containing 5 to be directly involved in Lp(a) uptake in VICs and valvular endothelial cells (VECs).^14^ Importantly, Lp(a) uptake was more pronounced in aortic valve cells compared to the hepatocellular carcinoma cell line HepG2,^14^ opening the exciting possibility for cardiac-specific targeting of Lp(a).

Despite recognizing that VECs are primarily exposed to circulating Lp(a), the direct effects of Lp(a) on VEC inflammation, metabolic reprogramming, and subsequent VIC activation remain unaddressed in the current study. Endothelial dysfunction and inflammation are early hallmarks of CAVD. Previous work revealed enhanced inflammation and transendothelial migration of monocytes in Lp(a)-stimulated human arterial endothelial cells (HAECs), predominantly orchestrated by Ox-PLs present on Lp(a). Addition of E06, a monoclonal antibody targeting Ox-PLs, abolished pro-inflammatory gene expression in HAEC,^15^ abrogated Lp(a)-induced osteogenic differentiation of VICs,^6,16^ and transgenic expression of a single-chain variable fragment of E06 (E06-scFv) in *Ldlr-*deficient mice ameliorated (among others) aortic valve stenosis, supporting the therapeutic potential of targeting Ox-PLs in CAVD. Concordantly, statin therapy, while effectively reducing LDL-cholesterol levels, confers no therapeutic efficacy in CAVD,^17^ mainly attributed to unaltered Lp(a) and Ox-PL levels. In a proof-of-concept Phase 2 trial, the antisense oligonucleotide IONIS-APO(a)Rx (now Pelacarsen) reduced plasma Lp(a) levels by >60%.^18^ Sourcing plasma samples from trial participants with the highest plasma Lp(a) levels (mean 445 nmol/L), Peletier *et al.*^7^ demonstrated a significant reduction in pro-inflammatory cytokine release from VICs when co-incubated with plasma obtained at Day 85 (peak therapeutic efficacy) compared to matched baseline samples. Although systemic inhibition of PFKFB3 was well tolerated and non-cytotoxic in a murine model of acute pancreatitis,^19^ valve-specific inhibition may be pivotal to achieve utmost efficacy in CAVD. Ongoing clinical trials may provide evidence for the benefits of lowering Lp(a) through Pelacarsen^20^ or targeted inhibition of PCSK9,^21,22^ although careful interpretation of the arising results may be warranted. While primary endpoints were specifically designed to evaluate the progression of AS, a follow-up period of 2–3 years may be insufficient to conclusively link lowering Lp(a) to attenuated AS progression. Systemic Lp(a) lowering in the at-risk population to delay disease onset, combined with inhibition of targeted (valve-specific) signalling pathways in advanced disease stages may provide a compelling path towards reducing the growing burden of CAVD.

Collectively, the current study by Peletier *et al*. provides a novel *in vitro* mechanism by which Lp(a) triggers valve inflammation and identifies PFKFB3 as a potential therapeutic target for CAVD. While clinical trials are underway to evaluate the impact of Lp(a) on AS progression, establishing the therapeutic potential of PFKFB3 inhibition for CAVD will require rigorous *in vivo* mechanistic studies to bridge the path to clinical translation.
